# Unraveling image construction by modality: Corpus-based diachronic insights into the reports to the Party Congress of China

**DOI:** 10.1371/journal.pone.0316017

**Published:** 2025-03-05

**Authors:** Jiannan Song, Dengcheng Li

**Affiliations:** School of English Studies, Xi’an International Studies University, Xi’an, Shaanxi, China; Kitami Institute of Technology, JAPAN

## Abstract

Recent years have witnessed a “discursive turn” in image construction studies. This article explores the linguistic features of image construction. We provide a corpus-based diachronic study of the distribution of modality in *The Reports to the 16*^*th*^ *to 20*^*th*^ *National Congress of the Communist Party of China* and unravel the role of the modal verbs in conceptualizing China’s images in *The Reports*. To analyze the national image, we need to understand how the various elements in *The Reports*, i.e., actors, actions, and situations, are not only depicted but also intricately interconnected in terms of volition, obligation, and prediction. The diverse national image profiles can be discerned through the lens of how the actors perceive the situations in relation to what is deemed desirable, committed, possible, important, and expected in the context of *The Reports*. The study finds that *The Reports*, based on the frequency, value, category and translation of the modal verbs, can project different image profiles of the nation, such as a reliable planner, a committed and powerful leader, and an active participant showing respect for others.

## Introduction

*The Report to the National Congress of the Communist Party of China* (hereafter, RNC) is a policy address delivered every five years by the General Secretary of the CPC. The RNC reviews China’s achievements over the past five years, analyzes the current domestic and international affairs, and outlines the major policies, goals, and roadmaps for China’s future development. In recent years, “the Chinese government has devoted much attention to constructing a positive image of the country, and particularly the promotion of Chinese ‘cultural soft power’” [1], and in return, a positive image of China can influence investor confidence and stimulate economic growth [2]. Because the RNC is an important platform for showing China’s image in the international community, the English version of the RNC is typically translated by Chinese translators selected and supported by the government. Therefore, the English translation of the RNC is an essential part of understanding the image of China and the Chinese government. However, the specific way in which the national image is constructed in the translation of the RNC remains unclear to academia. This study examines the linguistic representation of the national image in the context of the English translation of the RNC and reveals the messages aimed at the international community. This study is a diachronic analysis of the English translations of five RNCs over the past twenty years from the perspective of Systemic Functional Linguistics, with special attention to the choices of modal verbs and the nuances of the national image that arise from them. This study differs from the previous studies in several ways. First of all, previous research on the modality in political contexts has mainly focused on the image construct of political figures [3] and the translation strategies of political speeches [4–7], but the present study examines the relationship between the use of modal verbs and the construction of the national image. Secondly, this study explores the dynamic use of modal verbs from a diachronic rather than a synchronic perspective. Furthermore, we are concerned with the process of image construction based on the linguistic features in the RNCs, which is in contrast to most of the previous studies that view the national image construction as a socio-political phenomenon [8,9]. Based on the corpus of five RNCs, this study provides a linguistic analysis of modal verbs in the construction of the national image.

## Theoretical framework

### Image construction: Concept, function, and realization

Politically, the national image is formed through the collective perceptions and evaluations of a nation by the international community [9]. It is generally the aggregation of the direct and indirect experiences and emotions about the nation [10,11]. The construction of national image aims to exert a positive impact on a country’s reputation on its international relations [12]. In recent decades, interdisciplinary approaches to the national image have led to research in macro-areas such as economics [13], social psychology [14], political science [15], and communication [16]. Boulding [17] argues that the concept of national image is always used to describe the prestige of a country as constructed by both self-perception and the hetero-perception of other countries in international societies. It is worth noting that the views of social constructivism have found wide application in the study of the national image. Researchers believe that national image is not a fixed and stable fact, but rather a socially constructed flux shaped and influenced by social actors, cultural values, historical narratives, and power dynamics [18–22]. To date, research on China’s image has primarily focused on how China’s historical narrative, cultural diplomacy, and soft power strategies can be used to promote a particular aspect of the country’s image [23–25]. In the field of linguistics, the national image is analyzed in more detail, emphasizing the role of discursive acts and translational strategies in maintaining and reproducing power dynamics, ideologies, and narratives. On the one hand, strategies in discourse, such as metaphor [26], intertextuality [19], and evaluation [27], can reinforce or challenge existing beliefs or stereotypes about the national image by influencing how the rhetorical devices are used, how the narrative unfolds, and how the nation’s features are linguistically expressed and depicted. On the other hand, translation plays a significant role in shaping and transmitting national images, as it involves rendering texts and cultural elements from one language into another, which significantly influences how a nation is perceived and understood by the audience across linguistic and cultural boundaries [28–30].

### The force dynamics of modality

Halliday [31] argues that modality can express the speaker’s attitude, likelihood, permission, ability, or obligation regarding the action or state expressed by the main verb in a sentence. In other words, modality represents the intermediate degrees between positive and negative poles in a proposition or proposal, essentially forming a realm of uncertainty and dynamics in meaning. Halliday [31] categorizes the concept of modality into two groups, namely modalization and modulation. The former relates to the negotiation of probability and usuality, showing the speaker’s attitude about how possible or normal a particular action is in a proposition. The latter refers to the negotiation of obligation, commitment, and inclination, expressing the speaker’s attitude about how necessary or desirable a particular action is in a proposal [31,32]. By using the modality, a proposition or proposal can be evaluated or questioned in terms of the degree of credibility and commitment. There is a small class of verbs whose meaning is relative to modality, such as possibility or permission (*can, may*), obligation, inference or possibility (*must, should*), prediction, intention or hypothesis (*will, would*) [33,34]. The modal verbs can also be arranged on a semantic cline according to the degree of certainty or commitment. For example, “might*”,* and “could” suggest possibility or probability, but leave room for doubt or uncertainty, indicating a low level of certainty or commitment. “May” and “can” suggest a higher degree of certainty or commitment, indicating that something is very likely to happen, but there are still uncertainties. “Should” means a higher level of certainty or commitment, suggesting that something is expected, recommended, or morally/socially acceptable. “Must” enjoys the highest degree of certainty or commitment, meaning that something is required, legitimate, and logically necessary [35–37]. Given the polysemy of English modal verbs, we rely on the full specification of the meaning of modal verbs proposed by Coates [38]. Specifically, “must”, and “should” have the meaning of obligation/commitment and inference; “can” has the meaning of possibility, ability, and permission; “may” has the meaning of possibility and permission; “might” means possibility; “could” means possibility and hypothesis; “would” has the meaning of volition and hypothesis; “will”, and “shall” have the meaning of volition and prediction. When performing a modal analysis of the translated texts of the RNC, we test the different meanings of the same modal verb and select one or more meanings that best fit the context. The prevalence of certain modal verbs in the RNC translation may demonstrate the nation’s prioritization of specific virtues such as responsibility, commitment, or conviction.

In the context of linguistics and philosophy, the gap between the real world and the possible world can be bridged through modality. Depending on the provision of reliable evidence, the modality can have both subjective and objective meaning. The use of a modal verb can be objective if the proposition or proposal is based on highly reliable evidence, such as scientific data, proven facts, or well-established knowledge. For example, “the sun will rise in the east” is an objective proposition based on highly reliable evidence. Whereas, if the evidence is less reliable or if the speaker’s attitudes, beliefs, and intentions are involved, the use of a modal verb can become subjective. For instance, “The parcel might be received by now” involves speculation based on the speaker’s opinion regarding the situation [39,40]. Modality serves as a key indicator of a speaker’s attitude toward the objectivity of knowledge. Modal elements convey the evidential basis of a discourse, positioning propositions along a continuum of likelihood [41]. When modal verbs are objectified, they shift from expressing degrees of possibility to asserting certainty, and effectively transform a proposition or proposal into a fact and implying the inevitability of scenarios that have not yet occurred. In documents and action plans, modal verbs such as “will” and “shall” are used to convey certainty, which transforms hypothetical future scenarios into what appear to be definite outcomes. Such a choice for modality, which can be defined as the choice of “the mode within which an utterance is presented as true, reliable, and authoritative” [42:85], reflects a nation’s underlying mindset or belief system about its ability to foresee or control the future, revealing confidence, ambition, or a sense of inevitability in its planning.

### Research design

#### Research questions.

The purpose of this study is to examine the distribution of modal verbs in *The Reports to the 16*^*th*^ *to 20*^*th*^ *National Congress of the Communist Party of China* and to find out how the use of modal verbs conceptualizes China’s national image. The study aims to answer the following research questions:

1. How are the English modal verbs selected and distributed diachronically in the RNCs?
2. How do the frequency, value, category, and translation of modal verbs in the RNCs reveal the image of China?

#### Research corpus.

The corpus used in this study is from the authorized English publications of *The Reports of the 16*^*th*^*, 17*^*th*^*, 18*^*th*^*, 19*^*th,*^ *and 20*^*th*^ *National Congresses of the CPC* (2002-2022), which contain a total of 115,365 tokens (shown in Table 1). The reports highlight China’s achievements, challenges, and development plans and provide a platform to present the country’s priorities and strategies for the next five years. The reports generally consist of six sections based on the themes: Ideology, Economics, Politics, Culture, Diplomacy, and Party Building.

**Table 1:** The diachronic information of the RNCs corpus.

Corpus		Total size	Subcorpus size					
			Ideology	Economy	Politics	Culture	Diplomacy	Party Building
RNCs corpus	20^th^	25560	1779	1876	1185	1197	1160	2929
	19^th^	25402	3046	2110	1927	1389	1049	3325
	18^th^	22538	2506	2202	2351	1327	1098	3080
	17^th^	20932	2319	2542	2176	1297	1121	2673
	16^th^	20933	2456	3887	2663	1505	883	2668
	RNCs in total	115365	12106	12617	10302	6715	5311	14675

The diachronic RNCs corpus is used to demonstrate the development and changes in the use of modal verbs over the past two decades. In addition, the CROWN-Government (CRG), a contrastive corpus, is used to identify the statistically significant features of modal verbs in the RNCs. The CRG corpus is a subset of CROWN2021, a Brown-family American English corpus developed by the National Research Center for Foreign Language Teaching of Beijing Foreign Studies University (available at https://corpus.bfsu.edu.cn/info/1070/1335.htm). The CRG includes the government documents released in 2021 by the White House, federal departments, and state governments. The fact that the RNCs and CRG corpora belong to the same genre allows us to conduct a comparative analysis and identify the features of the modal verbs in the RNCs.

### Research procedures

We first use CLAWS4 to encode the modal verbs in the RNCs and then load them into AntConc 3.5.9 for quantitative analysis. After extracting the modal verbs and counting their frequencies in the RNC corpus, we compare them with the frequencies of modal verbs in the CRG corpus. A log-likelihood (LL) test is then conducted to find the overuse or underuse of modal verbs in the RNCs. The log-likelihood calculator is available at https://ucrel.lancs.ac.uk/llwizard.html. Rayson’s Log-Likelihood (LL) method [43] is a powerful statistical tool in corpus linguistics for identifying significant differences in word frequencies between two corpora. By comparing the observed frequency of a word in one corpus against its expected frequency, the LL method highlights patterns that reveal unique features or themes. Unlike tests that assume a normal distribution, LL is particularly suited to linguistic data, which often exhibits skewed distributions. This makes it an ideal choice for analyzing diverse text types. The method also employs thresholds of significance. A LL value of 3.84 or higher is significant at the level of p <  0.05 and of 6.63 or higher is significant at the level of p <  0.01. As the LL value increases, the frequency difference between the two corpora becomes larger. The minus sign is used before the LL value to indicate that the modal verbs are used less frequently in the RNCs than in the CRG. These characteristics have made LL indispensable for tasks such as keyword identification, genre analysis, and the exploration of discourse markers, and enable researchers to uncover meaningful insights into language use. Subsequently, we examine the diachronic use of modal verbs in the RNCs over the past twenty years, including critical comparisons every five years and general comparisons over twenty years. In addition, the use of modal verbs in each section of the RNCs is also analyzed diachronically and compared with the CRG corpus to identify the differences. Finally, taking modality dynamics into account, we address how the frequency, value, category, and translation of modal verbs contribute to the construction of the national image.

## Results and discussion

### The landscape of modal verbs in the RNCs and the CRG

Table 2 shows the comparison of the frequencies of the modal verbs in the RNCs and the CRG corpus. The two corpora differ in the total number of modal verbs and their categories. Although there are generally more modal verbs used in the RNCs than in the CRG corpus, the categories of modal verbs in the RNCs are much smaller than in the CRG corpus. The RNCs use “must”, “will”, “should”, and “can” as primary modal verbs, while the CRG has a more balanced selection of the modal verbs used in the corpus. Taking the modality value into account [30], we find that the frequency distribution of modality value in RNCs is “medium (n.f.=188.47)> high (n.f. =56.43)> low (n.f.=8.93)” and the CRG has a frequency distribution of “medium (n.f.=74.99)>low (n.f.=36.35)>high (n.f.=9.35)”. This implies that both the RNCs and the CRG prefer medium-value modal verbs, but they differ in the distribution of high and low-value modal verbs: the RNCs favor high-value modal verbs over low-value ones, while the CRG prefers low-value modal verbs to high-value ones. The LL test shows that the modal verbs occur more frequently in the RNCs (n.f. = 245.83) than in the CRG (n.f. = 120.69). Moreover, the overall frequency of high and medium-value modal verbs is significantly higher in the RNCs than in the CRG (LL = 230.59, p < 0.001; LL = 288.59, p < 0.001), and the overall frequency of low-value modal verbs in the RNCs is significantly less than in the CRG (LL = -133.28, p < 0.001). This also shows that the RNCs tend to overuse medium and high-value modal verbs and underuse low-value ones.

**Table 2:** The comparison of the modal verbs used in the RNCs and the CRG.

Modal verbs	The RNCs		The CRG		LL test	*p-*value
	a.f.	n.f. (‱)	a.f.	n.f. (‱)		
**High-value modality**						
must	651	56.43	45	9.35	230.59	*p* < .001
**Medium-value modality**						
will	1316	114.07	163	33.86	290.19	*p* < .001
should	760	65.88	74	15.37	211.33	*p* < .001
would	6	0.52	67	13.92	− 126.56	*p* < .001
shall	0	0	57	11.84	− 139.39	*p* < .001
*subtotal*	2082	188.47	361	74.99	288.59	*p* < .001
**Low-value modality**						
can	82	7.11	62	12.88	− 11.97	*p* < .001
may	19	1.65	77	16	− 106.04	*p* < .001
could	2	0.17	31	6.44	− 62.12	*p* < .001
might	0	0	5	1.04	− 12.23	*p* < .001
*subtotal*	103	8.93	175	36.35	− 133.28	*p* < .001
*total*	2836	245.83	581	120.69	282.97	p < .001

**Note:** a.f. = absolute frequency; n.f. = normalized frequency (per 10,000).

#### Example 1.

**The CRG:**

These public servants have significant experience in the agencies they will now temporarily lead. They will play an important role as the Biden administration prepares to coordinate a whole-of-government approach to tackle the challenges facing the nation, restore trust in our government, and ensure the federal government—and its many agencies—serves the American people. (Statements and Releases of the White House)

**The RNCs:**

**Source Text:** 我们**要**站稳人民立场、把握人民愿望、尊重人民创造、集中人民智慧,形成为人民所喜爱、所认同、所拥有的理论, 使之成为指导人民认识世界和改造世界的强大思想武器.

**Target Text:** We ***must*** stand firmly with the people, respond to their wishes, respect their creativity, and pool their wisdom to develop theories that they like, accept, and adopt and that become powerful tools guiding them in understanding and changing the world. (the 20^th^ RNC)

Example 1 illustrates the difference in the use of the high-value modal verb “must” in the 20^th^ RNC and the CRG. In the CRG, the Biden administration explains the basic responsibilities of the public servants and projects an image of an efficient and professional American government. The modal verb “must” is absent in this case, and the relationship between the public servants and their behaviors is not conceptualized as a matter of commitment and obligation but devotion and experience. English translations of Chinese official documents are usually prepared by professional teams linked to government bodies such as the Foreign Languages Press or the State Council Information Office. The teams prioritize conveying the original text’s tone and goals, often favoring global reception over literal translation. Shifts regularly occur in the English translation of Chinese political discourse [44]. In the 20^th^ RNC, the Chinese report describes the goals and the programs for the near future. The Chinese expression “我们要” literally means “we want” or “we wish”, but in the official translation, it is translated as “we must”. This reflects a deliberate choice to convey a sense of necessity and commitment. In Chinese official discourse, “要” often implies obligation or determination, especially in policy contexts. The use of “must” conveys the modal meaning of power, commitment, and inevitability. The choice of the high-value modal verb “must”*,* rather than the low or medium-value modal verbs such as “can”, “should”, and “will”, etc., emphasizes that the Chinese government not only has the strength and belief but also the determination to realize the stated goals. Translators opt for “we must” to match the intended tone of authority and resolve, ensuring that the message should be perceived as assertive and focused on action by an international audience.

In the case of the CRG, the construction of the national image is not fully attributed to the use of modality, but to the introduction of a third perspective (i.e., the public servants) and a positive prediction that the public servants can accomplish the objectives as required. In Table 2, the overuse of the modal verb “must” in the RNCs shows the fundamental identity of the government as a promoter and practitioner in the process of policy formulation and implementation. Halliday [30] points out that modal verbs with different values reflect the speaker’s degree of involvement in the formulation of a proposition or proposal. We can therefore assume that the overuse of the high-value modal verb “must” makes the national identity clearer in the RNCs by stressing the dominant role of the government in realizing the situations depicted and envisaged in the RNCs. The strong and consistent commitment strengthens the nation’s image as a responsible actor. The stated actions and measures proposed by the government are not speculation and suggestion, but conviction and obligation. This fosters the audience’s confidence in the country’s ability and strength to achieve its goals.

In the RNCs, the use of “will” reflects a sense of certainty and determination about China’s plans for the future, which suggests that the government is not just planning but is committed to seeing these plans through. This reinforces the image of a country with clear, structured, and dependable strategies. “Will” conveys an unambiguous direction, suggesting that the nation’s goals are firm and set to highlight the notion that China can be trusted to follow through on its developmental objectives. In addition, the use of “must” can be seen in areas where the country’s responsibilities or obligations are emphasized, further solidifying its role as a planner who is not only capable but also bound by duty to follow through on plans. The combination of “will” and “must” ensures that the image of China as a reliable planner is clearly conveyed through the RNCs.

### The diachronic synopsis of the modal verbs in the RNCs (16th-20th)

A corpus-based diachronic perspective unveils subtle variations in minor linguistic signs that provide important clues to broader contextual transformations [45]. From a diachronic perspective, we analyze the raw frequency of the modal verbs used in the RNCs over time (shown in Table 3). The LL test is conducted to examine the statistical significance of the interval changes and the overall changes of modal verbs from the 16^th^ to 20^th^ RNCs. The minus sign is used before the LL value to indicate that the modal verbs are used less frequently in one RNC than the other. Overall, there have been no significant changes in the past twenty years and the number of modal verbs remains relatively stable, except for the 18^th^ RNC, where there is a significant increase in the number of modal verbs from the 17^th^ to the 18^th^ RNC, (31.9%, p <  0.001), mainly due to the increased use of “should” during this period. However, significant changes can be observed if we consider the values of the modal verbs. Rather, high-value modal verbs decrease significantly by 35.3% (p < 0.001) and medium-value modal verbs increase significantly by 62.8% (p < 0.001). Low-value modal verbs show little numerical change over the past twenty years. A closer look at the diachronic changes of modal verbs reveals something interesting. The number of “must” decreases significantly from the 16^th^ RNC to the 20^th^ RNC (-35.3%, *p* < 0.001), but there is a temporary increase in the number of “must” between the 18^th^ RNC and the 19^th^ RNC (50.5%, p < 0.05).

**Table 3:** The diachronic analysis of the modal verbs in the RNCs (raw frequencies).

	2002 16th	2007 17th	% change 16th-17th	2012 18th	% change 17th-18th	2017 19th	% change 18th-19th	2022 20th	% change 19th-20th	% change 16th-20th
**High-value modality**										
must	167	113	− 32.3**	105	− 7.08	158	50.5*	108	− 31.7**	− 35.3 ***
**Medium-value modality**										
will	115	316	174.8***	53	− 83.2***	341	543.4***	491	44***	327 ***
should	206	8	− 96.1***	418	5125***	93	− 77.8***	35	− 62.4***	− 83 ***
would	2	3	50	0	− 100*	1	0	0	− 100	− 100
*total*	323	327	1.2	471	44.0 ***	435	− 7.6 **	526	20.9 **	62.8 ***
**Low-value modality**										
can	11	12	9.1	17	41.7	22	29.4	20	− 9.1	81.8
may	11	0	− 100***	2	0	1	− 50	5	400	− 54.6
could	0	0	0	1	0	0	− 100	1	0	0
*total*	22	12	− 45.5	20	66.7	23	15	26	13	18.2
**Total modality**										
*total*	512	452	− 11.72	596	31.9**	616	3.4	660	7.1	28.9

Note: Significance levels: ^* ^ =  *p* < 0.05, ^**^= *p* < 0.01, ^***^= *p* < 0.001.

**Fig 1:**
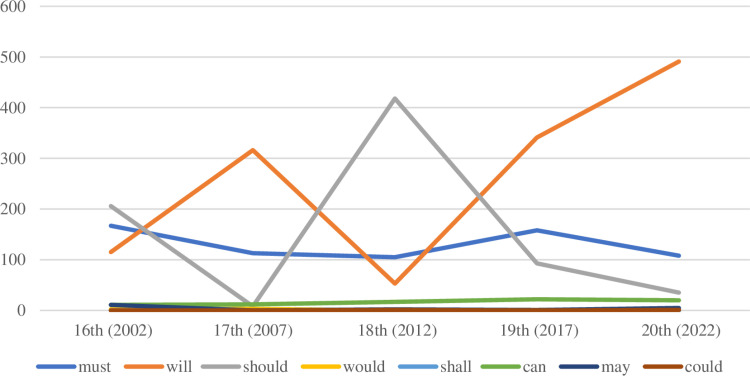
The diachronic changes of the modal verbs in the RNCs.

It is worth noting that there is a fluctuation in the number of “should” from the 16^th^ RNC to the 20^th^ RNC. The raw frequency of “should” drops from 206 in the 16^th^ RNC to 8 in the 17^th^ RNC, and then rises to 418 in the 18^th^ RNC, the highest number, before falling again to 93 in the 19^th^ RNC. In the 20^th^ RNC, the number of “should” is reduced to a minimum. Likewise, the number of “will” has changed dramatically over the past twenty years. The 16^th^ and 18^th^ RNCs have the lowest number of “will”, while, in the 17^th^ and 19^th^ RNCs, “will” is used much more frequently, and the number of “will” peaks in the 20^th^ RNC (Fig 1). In addition, there are no significant changes in the number of “would”, “shall”, “can”, “may”, and “could” over the twenty years. In the RNCs, “must” and “should” are mainly used to express the meaning of obligation/commitment, and “will” is primarily used to express volition and prediction, suggesting that the RNCs’ primary concerns are generally limited to commitment, will, and vision.

The usage of modal verbs in the RNCs over the past two decades provides valuable insights, revealing a clear pattern in how the country’s image is framed. By and large, the total number of modal verbs used in each of the five RNCs has remained relatively stable, but the selection of various modal verbs differs across the five RNCs. In the 16^th^ RNC, “should” appears most frequently, followed by “must”, with “will” being the least used. The modal verbs preferred by the 17^th^, 19^th^ and 20^th^ RNCs are almost identical: “will” stands out as the most frequently used, followed by “must” and “should” as the second and third choices, respectively. However, a noticeable difference occurs in the 18th RNC, where the frequency of “should” surpasses that of “will”. Since “should” conveys meanings of obligation, responsibility, and commitment, this change indicates a shift in the national image being projected by the 18th RNC. The heightened emphasis on “should” suggests that this period marks an effort to emphasize the responsible and committed nature of the government. The 18th RNC, therefore, focuses on portraying a more accountable and proactive government, ready to take on significant responsibilities. From the 19th to the 20th RNCs, there is a gradual but steady increase in the use of “will”, while the frequency of “should” declines. This shift is indicative of a return to a more assertive and determined stance in national discourse, with a clear emphasis on certainty, determination, and future-oriented planning. The increase in the use of “will” reflects the government’s desire to project confidence and clarity in policy formulation and execution, and signal a stronger resolve in navigating both domestic and international challenges. The evolution in the use of “will” and “should” over the years shows the changing dynamics of how China constructs its national image through these reports. The shift towards “will” in the more recent RNCs indicates a trend towards stronger willpower and scientific judgment in policy-making in response to the shifting domestic and international landscape since 2017. The evolution not only highlights a growing confidence in China’s policies but also a broader commitment to taking decisive action in shaping the country’s future. The method of constructing the national image through modal verbs has largely remained consistent, with a notable shift occurring in the 18^th^ RNC when the arrival of new central leadership brings changes in development direction, priorities, and communication likely in response to changes in the political, social, or international context at the time, or aimed at reinforcing specific aspects of the national image. Following the 18^th^ RNC, the use of modal verbs gradually reverts to previous patterns, suggesting that the overall construction of the national image becomes constant. In recent years, the continued high frequency of “will” in translations means that translators, in alignment with the nation’s objectives, have increasingly formed the preferred ways in which stress certainty and authority when presenting future plans and goals and highlight the reliability and stability of the national image.

#### Example 2.

**Source Text:** 完善中国特色现代企业制度, 弘扬企业家精神, 加快建设世界一流企业.

**Target Text:** We will improve the modern corporate system with distinctive Chinese features, adventure innovation, and move faster to help Chinese companies become world-class outfits.

(The 20^th^ RNC)

#### Example 3.

**Source Text:** 与时俱进加强军事战略指导, 高度关注海洋、太空、网络空间安全, 积极运筹和平时期军事力量运用, 不断拓展和深化军事斗争准备, 提高以打赢信息化条件下局部战争能力为核心的完成多样化军事任务能力.

**Target Text:** We should attach great importance to maritime, space, and cyberspace security. We should make active planning for the use of military forces in peacetime, expand and intensify military preparedness, and enhance the capability to accomplish a wide range of military tasks, the most important of which is to win local war in an information age.

(The 18^th^ RNC)

Excerpted from the 20^th^ RNC, Example 2 concerns the measures to promote a high-level socialist market economy. There are two differences between the source text and the target text: the use of the modal verb and the collocation of the modal verb. The original Chinese text has neither a subject nor a modal verb. However, the modal verb “will” is added to the English text, bringing a sense of volition and prediction to the proposition. Meanwhile, the target text adds a subject “we” to collocate with “will” to clarify that the nation is an active implementer of the economic programs and to emphasize the role of the nation in taking actions to support business development, increasing the certainty or predictability in the future. Example 3 excerpts from the 18^th^ RNC and concerns the national plans and programs to improve the military capacities. Similarly, the source text in Example 3 has no subject and the modal verbs are missing. In the target text, the modal verb “should” and the subject “we” are added to emphasize the nation’s commitment to developing the military force. The modal verb “should” relates to social norms and duties, indicating the expected or preferred behavior within a given context or society. The use of “we will” and “we should” contribute to creating a collective identity by upholding common values and goals for the development of the nation. Constructivism posits that texts play a key role in constructing a national image as they involve the use of language, narratives, and symbols to shape perceptions of the nation [46]. Drawing upon the modal verbs, individuals and groups engage in the process of meaning-making, framing, and interpretation that contribute to the formation and maintenance of a national image. The presence of modal verbs in a text indicates the speaker’s attitude, evaluation, or belief in the truth value of the proposition or proposal. According to Halliday and Matthiessen [47], “will” conveys the speaker’s wishes or expectations regarding the provision of goods and services, and “should” functions as a directive or command to the audience. Therefore, the use of “will” and the use of “should” invoke two different national images: the first sees the nation as a reliable planner whose plans and actions proposed in the RNCs have been analyzed and taken into account the social norms and conditions, and the second sees the nation as a committed leader, whose plans and measures proposed in the RNCs are motivated by commitments to the country.

The image of China as a committed and powerful leader is realized primarily through the use of modal verbs such as “must” and “should.” The modal verbs serve to express obligation, authority and responsibility. “Must” in particular carries a strong sense of imperative, which suggests that the Chinese government views itself as a leader who is not merely engaged in passive planning but is actively driving its objectives forward with a sense of necessity and power. The government has the authority to make decisions that are binding and critical for the country’s future. Meanwhile, the use of “should” in the RNCs implies a commitment to certain goals or behaviors, which indicates that China sees itself as a leader with a moral or strategic responsibility to shape the future in a positive direction. The language of “must” and “should” in this context reflects a leader who is powerful and ethically committed to fulfilling its role.

### The diachronic distribution of main modal verbs in each section of the RNCs

Table 4 provides an overview of the diachronic distribution of the four main modal verbs “must”, “should”, “will”, and “can” in the different sections of the RNCs (excluding “shall”, “could”, “would”, and “may” due to lack of use)*.* Using the CRG as a reference corpus, the log-likelihood test is applied to examine the overuse or underuse of the modal verbs across the six major sections of the RNCs: “Ideology”, “Economy”, “Politics”, “Culture”, “Diplomacy” and “Party Building”. Table 4 shows that in the six sections, the main modal verbs are not distributed in the same way. Instead, the “Ideology” section and the “Party Building” section have the greatest tendency to overuse “must” (LL = 272.2, p < 0.01; LL = 181.8, p < 0.01), the “Economy” and the “Politics” sections are more inclined to overuse “should” (LL = 210.1, p < 0.01; LL = 148.0, p < 0.01) and the “Culture” and the “Diplomacy” sections are more likely to overuse “will” (LL = 131.8, p < 0.01; LL = 55.5, p < 0.01).

**Table 4:** Diachronic distribution of the main modal verbs in the different sections of the RNCs.

The RNCs	Ideology		Economy		Politics		Culture		Diplomacy		Party Building	
	n	LL	n	LL	n	LL	n	LL	n	LL	n	LL
**Must**												
20^th^-2022	23	69.6 **	6	5.9 *	3	2.1	2	0.5	0	−2.1	31	79.8 **
19^th^-2017	48	147.6 **	8	9.6 **	8	10.7 **	5	5.8 *	0	−1.9	28	62.2 **
18^th^-2012	22	52.0 **	6	4.6 *	8	8.4 **	3	1.7	0	−2.0	12	14.4 **
17^th^-2007	24	62.9 **	6	3.6	22	57.4 **	11	27.0 **	0	−2.1	16	28.9 **
16^th^-2002	26	68.5 **	42	104.4 **	13	19.8 **	18	53.2 **	0	−1.6	34	97.2 **
total	143	272.2 **	68	82.8 **	54	68.5 **	39	59.6 **	0	−9.4 **	121	181.8 **
**Should**												
20^th^-2022	14	21.6 **	1	−1.6	0	−3.6	1	−0.5	0	−3.5	9	3.2
19^th^-2017	11	5.7 *	7	3.1	18	32.1 **	6	4.5 *	8	12.3 **	26	37.7 **
18^th^-2012	19	27.6 **	62	207.2 **	54	164.0 **	38	135.5 **	7	8.9 **	69	199.1 **
17^th^-2007	0	−7.0 **	0	−7.6 **	0	−6.5 *	0	−3.9 *	7	8.7 **	0	−8.0 **
16^th^-2002	12	10.5 **	75	194.1 **	32	66.8 **	17	35.8 **	12	29.6 **	29	56.4 **
total	56	35.2 **	145	210.1 **	104	148.0 **	62	92.3 **	34	38.0 **	133	156.2 **
**Will**												
20^th^-2022	3	−1.8	65	166.7 **	30	64.9 **	43	116.8 **	4	0.0	73	144.6 **
19^th^-2017	10	0	58	127.3 **	19	14.8 **	35	74.7 **	11	9.6 **	54	74.1 **
18^th^-2012	3	−4.6 *	3	−3.3	2	−6.2 *	6	0.4	19	30.1 **	4	−5.0 *
17^th^-2007	14	3.7	66	136.7 **	22	17.6 **	23	37.0 **	15	18.1 **	52	86.0 **
16^th^-2002	10	0.3	7	−3.3	26	19.5 **	1	−4.8 *	13	17.6 **	10	0.1
total	40	0	199	203.2 **	99	59.5 **	108	131.8 **	62	55.5 **	193	157.0 **
**Can**												
20^th^-2022	7	5.9 *	1	−1.0	2	0.1	0	−3.0	0	−3.0	2	−1.0
19^th^-2017	5	0.3	0	−5.3 *	5	1.9	1	−0.4	2	0.3	3	−0.4
18^th^-2012	4	0.2	2	−0.3	0	−5.9 *	0	−3.4	1	−0.1	4	0.0
17^th^-2007	1	−1.7	1	−2.1	1	−1.5	0	−3.3	1	−0.1	0	−6.7 **
16^th^-2002	2	−0.5	3	−0.9	1	−2.3	0	−3.8	1	0.0	0	−6.7 **
total	19	0.5	7	−5.6 *	9	−1.3	1	−10.1 **	5	−0.5	9	−5.2 *

Note: Significance levels: ^* ^ =  *p* < 0.05, ^**^= *p* < 0.01.

The distribution of modal verbs across the different sections varies according to the different periods. Comparing the frequencies of “must”, “should”, “will”, and “can” in the 20^th^ RNC to those in the 16^th^ RNC, we find that the frequency of “must” in the “Ideology” and “Party Building” sections has been stable and remains at a high level of significance. However, “must” is less frequently used in the “Economic” section (LL value drops dramatically from 104.4 to 5.9), the “Politics” section (LL value drops dramatically from 19.8 to 2.1), and the “Culture” section (LL value drops dramatically from 53.2 to 0.5). Interestingly, although “must” is overused in almost all sections of the RNCs, in the “Diplomacy” section, the frequency of “must” is 0 (LL = -9.4, p < 0.01) across different periods. The absence of “must” in this section may indicate that in line with its peaceful development strategy, China addresses international problems in a non-intrusive manner. Unlike the strong, certain tones found in other sections, the lack of modal verbs suggests that China’s approach to diplomacy is more adaptable and flexible. It implies that diplomatic outcomes are not fixed or guaranteed, but depend on changing global circumstances, international cooperation, and external factors. By avoiding modal verbs, the government may be signaling that foreign policy needs to remain open-ended and responsive to these variables, rather than being framed as an absolute or rigid plan. The frequency of “should” has been increasing in the “Ideology” section over time (LL value increases dramatically from 10.5 to 21.6) except for the 17^th^ RNC, where the frequency of “should” falls to zero. The fact that the “Ideology” has an increasing preference for “should” suggests that China is committed to the ideological construction that guides the country’s development and feels obliged to follow the ideological path. The distribution of “will” varies by different sections. In the “Ideology” section, the frequency of “will” shows no significant difference compared to that of the CRG, and in the 18^th^ RNC, “will” is underused, suggesting that “will” is not the most prominent modal verb in the “Ideology” section. In contrast, from the 16^th^ to 20^th^ RNCs, the increasing overuse of “will” in the “Economic”, “Politics”, “Culture”, and “Party Building” sections (LL value rises from -3.3 to 166.7, from 19.5 to 64.9, from -4.8 to 116.8, and from 0.1 to 144.6, respectively) can be observed. However, the frequency of “will” decreases in the “Diplomacy” section (LL value drops dramatically from 17.6 to 0. “Can” is only used frequently in the “Ideology” section of the 20^th^ RNC, and is considered a marginal modal verb in other sections of the RNCs across different periods. This implies that “can” is not an important modal verb in the RNCs. The contrast between the assertive tone in the “Ideology” and “Party Building” sections and the more flexible tone in the “Diplomacy” section highlights different approaches to domestic policy and foreign relations. While the government emphasizes certainty and commitment in internal matters, it adopts a more adaptable stance in diplomacy, balancing domestic assertiveness with international flexibility. China’s image as an active player showing respect for others is actualized in the diplomatic sections of the RNCs through the use of modal verbs like “should” and “will.” “Should” suggests mutual obligation, reflecting China’s commitment to respectful, goal-oriented relationships with other nations, while “will” emphasizes flexibility and willingness to cooperate. The fewer usage of “must” highlights China’s diplomatic approach which values respect, collaboration, and adaptability in international relations.

#### Example 4.

**Ideology**

**Source Text:** 我们**要**坚持对马克思主义的坚定信仰、对中国特色社会主义的坚定信念, 坚定道路自信、理论自信、制度自信、文化自信.

**Target Text:** We *must* remain firm in our conviction in Marxism and socialism with Chinese characteristics and strengthen our confidence in the path, theory, system, and culture of socialism with Chinese characteristics.

(the 20^th^ RNC)

#### Example 5.

**Economy**

**Source Text:** 我们**要**坚持以推动高质量发展为主题, 把实施扩大内需战略同深化供给侧结构性改革有机结合起来, 增强国内大循环内生动力和可靠性.

**Target Text:** Pursuing high-quality development as our overarching task, we *will* make sure that our implementation of the strategy to expand domestic demand is integrated with our efforts to deepen supply-side structural reform.

(the 20^th^ RNC)

#### Example 6.

**Diplomacy**

**Source Text:** 倡导构建人类命运共同体, 促进全球治理体系变革。我国国际影响力、感召力、塑造力进一步提高, 为世界和平与发展作出新的重大贡献.

**Target Text:** China champions the development of a community with a shared future for mankind, and has encouraged the evolution of the global governance system. With this we have seen a further rise in China’s international influence, ability to inspire, and power to shape; and China has made great new contributions to global peace and development.

(the 19^th^ RNC)

The analysis presented in Table 4 shows that the choice and use of the modal verbs vary depending on the topics. The high-value modal verb “must” is used more frequently in the “Ideology”, “Politics” and “Party Building” sections, but very rarely in the “Diplomacy” section, where “will” and “should” are preferred. Moreover, the “Economy” section is more inclined to use “should”. To illustrate the different uses of “must”, “should”, and “will”, we use Examples 4 to 6 to show the usage of the modal verbs in the “Ideology”, “Economy” and “Diplomacy” sections. The original Chinese texts of Example 4 and Example 5 have the same structure “我们要”, which means “we want”, or “we wish”, and in the English texts, it is translated as “we must” in the “Ideology” section and “we will” in the “Economy” section. “We” refers to the actor who plays an active role in action and is often used to express a sense of inclusion in particular contexts. The use of “we will” and “we must” include not only the government but also the Chinese people, meaning that the government and the people are closely related and belong to the same community. Li [48] points out that the use of the different degrees of modal verbs can expand the space of dialogue and take into account other viewpoints and options. The high-value modal verb “must” means the narrowing of the dialogue space in which there is no room for a different opinion. The difference in the choice of modal verbs between “must” and “will” indicates the different profiles of the country’s image. Issues of ideology and Party-building are a high priority for the country. The use of “must” suggests that in terms of these key issues, the country plays a dominant and authoritarian role in carrying out the essential tasks, and underscores the government’s deep commitment as a powerful leader. In the economic field, the use of “will” weakens the involvement of the government in economic activities but emphasizes the government’s identity as a rational analyzer, whose views of economic development are based on the inferences and predictions of the relevant social facts. Taking the 20^th^ RNC as an example, the use of “will” is much greater than that of “must”, which to some extent reflects the fact that the market economy with Chinese characteristics continues to develop profoundly and that the government has become an advisor rather than a player in economic activities. Table 4 shows that “must” is always missing in the “Diplomacy” section of the RNCs in different periods, but “will” and “should” are often used, reflecting that China hopes to project an unintentional, and non-interventionist player who shows respect for others in the diplomatic arena. Therefore, in Example 6, neither the Chinese text nor the English text contains the modal verbs, and this makes the “Diplomacy” section in the RNC a factual statement that is free of attitudes or judgments from the Chinese government.

## Conclusion

Image construction is an important part of political discourse, as well as one of the main reasons for the selection and use of modal verbs. In the RNCs, the image construction relies on how the actors, actions, and situations are conceptualized and interrelated in terms of volition, obligation, and prediction. The diverse national image profiles can be discerned through the lens of how the actors perceive the situations in relation to what is deemed desirable, committed, possible, important, and expected in the context of the RNCs. This study provides a corpus-based examination of the distribution of the main modal verbs in the English translations of the RNCs over different periods. It explores how the national images are ritually represented through modal verbs and discusses the effects of the modal verbs on the image construction. The selection of modal verbs in the RNCs can indicate the nation’s commitment to certain values, the way it perceives authority, and its willingness to engage in certain actions or obligations. The study shows that the RNCs use modal verbs more frequently than the CRG. From a diachronic perspective, the way the RNCs use modal verbs has been largely consistent over the past 20 years. The distribution of the main modal verbs varies according to the different sections of the RNCs. For example, the “Ideology”, “Policy” and “Party Building” sections use “must” more frequently, while “must” is rarely used in the “Diplomacy” section. Based on the frequency, value, category, and translation of the modal verbs, the RNCs can project different image profiles of China. In this study, we find that the image of China in the RNCs is conceptualized as a reliable planner, a committed and powerful leader, and an active participant showing respect for others. A limitation of this study is that it focuses mainly on modality, without fully exploring how it interacts with other rhetorical strategies such as metaphor and framing, which are also important for image construction. Future research can address this gap by examining these strategies alongside modality, providing a more comprehensive understanding of how China’s image is shaped in political discourse [44,45]. Furthermore, the study does not consider how international audiences perceive the modal verbs used in the RNCs. Exploring their perceptions will offer valuable insights into how China’s image is projected and received globally.

## Supporting information

S1 File Supporting information. (ZIP)
